# Predictors of circulating INTERLEUKIN-6 levels in head and neck cancer patients

**DOI:** 10.1186/s41199-018-0029-5

**Published:** 2018-05-28

**Authors:** Sylvine Carrondo Cottin, Stéphane Turcotte, Pierre Douville, François Meyer, Isabelle Bairati

**Affiliations:** 1 Centre de recherche sur le cancer, Université Laval, 6, rue McMahon, 1899-2, Quebec City, QC G1R 2J6 Canada; 2 Centre de recherche du CHU de Québec - Université Laval, Quebec City, QC Canada

**Keywords:** Head and neck cancer, Interleukin-6, Tobacco use, Alcohol consumption

## Abstract

**Background:**

Circulating interleukin-6 (IL-6) improves outcome prediction for second primary cancer (SPC) in head and neck cancer (HNC) patients. This study aimed to identify factors associated with IL-6 serum levels in HNC patients.

**Methods:**

This study was conducted as part of a phase III chemoprevention trial. IL-6 was measured using chemiluminescent immunometric assay on pretreatment serum sample obtained from 527 stage I-II HNC patients. Patients’ lifestyle habits, sociodemographic, medical and tumor characteristics were evaluated before radiation therapy (RT). Factors independently associated with IL-6 levels before RT were identified using multiple linear regression.

**Results:**

The median IL-6 serum level was 3.1 ng/L. In the multivariate analysis, eight factors were significantly associated (*p* <  0.05) with IL-6: age, gender, marital status, body mass index, tobacco consumption, comorbidities, Karnofsky Performance Status and HNC site. Smoking duration and lifetime pack-years were positively associated with IL-6 serum levels in a dose-response relationship (*p*-value for trend ≤0.03).

**Conclusions:**

Circulating IL-6 is a strong predictor of the occurrence of SPC in HNC patients. We identified eight factors independently associated with serum IL-6 levels in 527 stage I-II HNC patients.

The dose-response relationship between lifetime smoking and IL-6 serum levels suggested a causal role of tobacco exposure on IL-6 production. Further studies are needed to establish whether the effect of tobacco exposure on SPC could be partly mediated by IL-6, a pro-inflammatory cytokine.

## Background

Head and neck cancers (HNC) are the seventh most common cancer worldwide with an annual incidence of 15 per 100,000 [1]. Due to modern surgery and radiation therapy, locoregional control of early-stage HNC has improved to reach 50 to 95% at 5 years depending on the cancer site [2]. In particular, early-stage glottic cancer has an excellent prognosis with cause-specific survival varying between 88 and 97% at 5 years [3]. However, patients with a history of HNC are at high risk for developing second primary cancers (SPC) [4] as, within 5 years, about 20% will be diagnosed with one or more multiple primary cancers, which strongly compromises their survival.

Interleukin-6 (IL-6) is a pro-inflammatory cytokine that has been described as being involved in many tumorigenesis processes [5], particularly angiogenesis [6, 7], tumor cell migration and invasion [8] and cell growth and proliferation [9, 10]. In a recent cohort study conducted among 6545 middle-aged adults, IL-6 serum level was able to predict all-causes and cancer-related mortalities over a period of 17 years [11]. In addition, when the three promising inflammatory biomarkers (α1-acid glycoprotein, C-reactive protein and IL-6) were considered together, IL-6 was the only one that remained associated with mortality by cancer. Strong evidence from a meta-analysis also showed that higher IL-6 serum levels were associated with poor prognosis in non-small cell lung cancer patients [12]. In HNC, IL-6 was identified as a predictor of recurrence and overall survival in a cohort of 444 HNC patients, most of whom had pharyngeal and advanced cancers [13]. We also reported that higher pre-treatment IL-6 serum levels were significantly associated with the occurrence of SPC [14] and death by SPC in 527 early-stage HNC patients.

Heavy smoking and alcohol consumption are standard risk factors for HNC [15]. In addition, smoking status affects SPC risk and site [16]. Associations between IL-6 serum levels and both tobacco and alcohol consumption have been described in various populations [17, 18]. A cross-sectional study conducted on 444 HNC patients at the time of their diagnosis suggested that only two health behaviors (smoking and decreased sleep) were independently associated with IL-6 serum levels [19]. As IL-6 appears to be a valid predictor in cancer prognosis, it would be useful to further examine the effects of cumulative past exposure to various health behaviors on IL-6 serum levels. The objective of this study was to identify the predictors of IL-6 serum levels in HNC patients. In addition, we specifically examined the influence of long-term tobacco and alcohol consumption on IL-6 serum levels.

## Methods

### Study population

This study was conducted as part of a randomized controlled chemoprevention trial (NCT00169845) [20] evaluating the efficacy of α-tocopherol and β-carotene supplementation in reducing the incidence of SPC. The ethics review board of the five radiotherapy centers participating in the trial approved the protocol. All patients gave their written informed consent prior to randomization.

Eligible patients were aged 18 years and over, had received a first diagnosis of stage I or II squamous cell carcinoma of the head and neck area (tongue, gums or mouth, oropharynx, hypopharynx and larynx) and were treated by radiation therapy (RT) between October 1, 1994 and June 6, 2000 in one of five radiotherapy centers in the province of Quebec, Canada. A total of 527 patients among the 540 HNC randomized patients (97.6%) with available pretreatment serum samples were included in the analyses.

### Baseline data collection

All the baseline data were collected before the patients were randomized in the trial and before they started their RT. Trained research nurses administered a structured general questionnaire to evaluate patients’ characteristics, including their demographic and socioeconomic data, Karnofsky Performance Status (KPS) [21] and medical history. The Charlson Comorbidity Index, a comorbidity score, was calculated based on the medical history [22]. Sleep disturbance was assessed using the item 11 of the EORTC QLQ-C30 instrument [23]. The study nurses weighed and measured the patients during the visit and asked patients about their weight at 20 years of age. Current body mass index (BMI) and BMI at 20 years of age were calculated.

A validated food frequency questionnaire [24] assessing dietary intake over the year preceding randomization was used to evaluate the self-reported consumption of beer, wine, aperitifs and spirits. A standard drink was pre-defined for beverages (beer: 1 bottle; wine: 4 oz; aperitif: 2 oz; spirits: 1 oz) and 10 profiles of consumption were proposed (number of drinks per day, week or month). Average daily intake of alcohol (g per day) was then calculated. The same method was used to assess the average daily intake of alcohol over the past 10 years. In addition, a question assessed whether patients had a history of alcohol abuse.

Lifetime smoking habits were recorded for all tobacco products (cigarette, cigar, pipe, chewing) using a structured questionnaire. For all products and for all smoking periods, we assessed the mean consumption per day, week or month and the duration. In addition, age at first cigarette, time since quitting cigarette smoking and patients’ current cigarette smoking status were recorded. The number of pack-years, the duration and intensity of cigarette smoking as well as the number of years since quitting cigarette smoking were calculated.

The radiation oncologists provided detailed information on the primary tumor: precise site, dimensions and clinical stage according to the TNM classification [25].

### IL-6 measurement

Baseline serum samples were obtained at the time of randomisation before any trial intervention and before RT. Samples were kept frozen at − 80 °C and then thawed shortly before determination of IL-6 levels. In order to avoid any influence of the trial intervention, IL-6 was measured on baseline samples using an IMMULITE®1000 immunoassay analyzer with chemiluminescent immunometric assay (Siemens Healthcare Diagnostics).

### Statistics

IL-6 serum level was the outcome and was treated as a continuous variable. Given the skewed distribution of IL-6, IL-6 was log transformed (natural log) for all analyses. The selected sociodemographic factors were sex, age (≤ 50, 51–60, 61–70 and >  70), marital status (not married vs. married) and household income (< $40,000 vs. ≥ $40,000). Clinical variables considered were KPS (60–80 vs. ≥ 90), Charlson Comorbidity Index (0, 1, ≥ 2), diabetes (yes vs. no), cancer site (glottis vs. other) and TNM stage (stage I vs. II). The selected health behavior variables were BMI, past and current tobacco and alcohol consumption. Current BMI and BMI at 20 years of age were classified according to the WHO categories (< 18.5: underweight; ≥ 18.5 to < 30: normal weight and overweight; ≥ 30: obesity) [26]. History of cigarette smoking was considered using variables measuring the duration of cigarette smoking, the average number of cigarettes smoked and the average number of pack-years consumed. These variables were categorized according to the quartiles in those exposed to cigarette smoking. Alcohol consumption during the preceding year (g/day in quartiles), alcohol consumption in the past 10 years (g/day in quartiles) as well as a history of alcohol abuse (yes vs. no) were considered.

Student *t*-test or analysis of variance were performed to examine all the associations between IL-6 serum levels and the selected factors. All the variables associated with IL-6 serum levels in the bivariate analyses (*P* ≤ 0.20) were considered for inclusion in a multivariate linear regression model. The final model was constructed using a backward elimination procedure. Before fitting the final model, tests for collinearity among the variables were done. The regression diagnostics methods used on the final model showed the appropriateness of the regression assumptions (linearity, variance homogeneity, Gaussian distribution of the residuals). A non-parametric bootstrap resampling method was performed on the residuals to assess the reliability of the regression model [27]. The bootstrap validation process began by forming 2000 bootstrap samples of equal size (*n* = 527) with replacement. Each bootstrap sample was used as a training sample. Regression coefficients were estimated by the bootstrapping method and their 95% confidence intervals were calculated using the method of bias-corrected accelerated percentile intervals.

For all variables describing lifetime cigarette smoking, the associations with IL-6 were tested using a multivariate linear regression adjusting for all factors retained in the final model. Tests for linear trend were done to verify whether IL-6 serum levels tended to increase with cumulative past exposure to cigarette consumption. For trend tests, exposures were considered as linear for ordered categorical variables.

All statistical analyses were performed using SAS (version 9.4; SAS Institute Inc.) and R (version 3.4.0; R development Core Team) statistical software. All statistical tests were 2-sided (α = 0.05).

## Results

The median baseline IL-6 serum level was 3.1 ng/L (interquartile range: 2.2–4.4). Characteristics of the 527 HNC patients are presented in Table 1. The mean age was 62.5 years old (SD = 9.8) and most of the patients were male (78.8%). Patients’ HNC were predominantly stage I (61.7%) glottic cancer (64.5%). Only 3.4% of patients were underweight, while 17.8% were obese. Use of tobacco products during the previous year was reported by 70.6% of the HNC patients, while 4.9% reported never having consumed tobacco products during their life. Daily alcohol consumption during the previous year was low (the median value was 2.32 g/day) and 16.7% reported having a history of alcohol abuse.

**Table 1:** Sociodemographic, clinical and health behavior characteristics of patients with head and neck cancer (*N* = 527)

Factors	Categories	N (%)
Age (years)	≤ 50	55 (10.4)
	> 50 ≤ 60	155 (29.4)
	> 60 ≤ 70	197 (37.4)
	> 70	120 (22.8)
Sex	Female	112 (21.3)
	Male	415 (78.8)
Current body mass index (kg/m^2^)	< 18.5	18 (3.4)
	≥ 18.5 < 30	415 (78.8)
	≥ 30	94 (17.8)
Body mass index at 20 years (kg/m^2^)	< 18.5	47 (8.9)
	≥ 18.5 < 30	462 (87.7)
	≥ 30	18 (3.4)
Marital status	Not married	137 (26.0)
	Married	390 (74.0)
Household income (per year)	< $40,000	388 (73.6)
	≥ $40,000	139 (26.4)
Karnofsky Performance Status (%)	90–100	485 (92.0)
	60–80	42 (8.0)
Charlson Comorbidity Index	0	323 (61.3)
	1	128 (24.3)
	≥ 2	76 (14.4)
Diabetes	No	488 (92.6)
	Yes	39 (7.4)
Glottic cancer	No	187 (35.5)
	Yes	340 (64.5)
TNM clinical stage	I	325 (61.7)
	II	202 (38.3)
Tobacco consumption (all tobacco products)	Never	26 (4.9)
	Former	372 (70.6)
	Current	129 (24.5)
History of alcohol abuse	No	439 (83.3)
	Yes	88 (16.7)
Sleep disturbance	Not at all	285 (54.1)
	A little	132 (25.0)
	Quite a bit	62 (11.8)
	Very much	48 (9.1)

Unadjusted associations between IL-6 levels and the selected factors are presented in Table 2. In these analyses, 14 factors were associated with IL-6 (*P*-value ≤0.20): age (*P* <  0.0001), sex (*P* = 0.04), current BMI and BMI at 20 years of age (*P* = 0.02 and 0.04, respectively), marital status (*P* = 0.02), household income (*P* = 0.02), KPS (*P* <  0.0001), Charlson Comorbidity Index (*P* <  0.0001), diabetes (*P* = 0.06), cancer site (*P* = 0.0004), TNM stage (*P* = 0.007), tobacco consumption (*P* = 0.0008), history of alcohol abuse (*P* = 0.03) and sleep discturbance (*P* = 0.20).

**Table 2:** Associations between selected factors and IL-6 serum levels (*N* = 527)

Factors	Categories	Median IL-6 (ng/L) (IQR)	Crude β (SE)	*P*-value
Age (years)	≤ 50	2.4 (1.0–3.7)	Reference	
	> 50 ≤ 60	2.9 (2.0–3.9)	0.15 (0.11)	
	> 60 ≤ 70	3.2 (2.4–4.4)	0.34 (0.11)	
	> 70	3.5 (2.6–5.1)	0.48 (0.11)	< 0.0001
Sex	Female	3.0 (2.2–3.9)	Reference	
	Male	3.1 (2.2–4.6)	0.15 (0.07)	0.04
Current body mass index (kg/m^2^)	< 18.5	3.5 (2.5–5.1)	0.13 (0.17)	
	≥ 18.5 < 30	2.9 (2.1–4.1)	Reference	
	≥ 30	3.8 (2.7–4.9)	0.22 (0.08)	0.02
Body mass index at 20 years (kg/m^2^)	< 18.5	3.0 (2.0–4.9)	−0.06 (0.11)	
	≥ 18.5 < 30	3.1 (2.2–4.4)	Reference	
	≥ 30	4.1 (2.7–7.0)	0.43 (0.17)	0.04
Marital status	Not married	3.3 (2.5–4.6)	Reference	
	Married	3.0 (2.1–4.4)	−0.16 (0.07)	0.02
Household income (per year)	< $40,000	3.3 (2.3–4.6)	Reference	
	≥ $40,000	2.7 (2.0–3.7)	−0.22 (0.07)	0.02
Karnofsky Performance Status (%)	90–100	3.0 (2.2–4.3)	Reference	
	60–80	4.9 (2.9–5.8)	0.46 (0.11)	< 0.0001
Charlson Comorbidity Index	0	2.9 (2.1–3.9)	Reference	
	1	3.4 (2.1–5.1)	0.24 (0.07)	
	≥ 2	3.7 (2.8–5.2)	0.38 (0.09)	< 0.0001
Diabetes	No	3.0 (2.2–4.4)	Reference	
	Yes	3.6 (2.8–5.0)	0.22 (0.12)	0.06
Glottic cancer	No	3.5 (2.5–5.0)	Reference	
	Yes	2.9 (2.1–4.1)	−0.22 (0.06)	0.0004
TNM clinical stage	I	2.9 (2.1–4.2)	Reference	
	II	3.4 (2.4–4.9)	0.17 (0.06)	0.007
Tobacco consumption (all tobacco products)	Never	2.2 (1.0–3.3)	Reference	
	Former	3.0 (2.2–4.4)	0.48 (0.14)	
	Current	3.5 (2.4–5.1)	0.57 (0.15)	0.0008
History of alcohol abuse	No	3.0 (2.2–4.3)	Reference	
	Yes	3.6 (2.5–5.3)	0.18 (0.08)	0.03
Alcohol consumption during the preceding year (g/d)	0	3.4 (2.3–4.6)	Reference	
	≤ 2.32	3.0 (2.1–4.3)	−0.03 (0.09)	0.31
	> 2.32 ≤ 12.48	2.9 (2.2–4.2)	−0.13 (0.09)	
	> 12.48	3.1 (2.4–5.0)	0.02 (0.09)	
Alcohol consumption during the past 10 years (g/d)	≤ 0.39	3.3 (2.1–4.3)	Reference	
	> 0.39 ≤ 10.49	3.1 (2.2–4.4)	−0.05 (0.09)	
	> 10.49 ≤ 42.88	2.8 (2.1–4.1)	−0.08 (0.09)	
	> 42.88	3.3 (2.5–5.1)	0.04 (0.09)	0.50
Sleep disturbance	Not at all	3.0 (2.1–4.4)	Reference	
	A little	3.0 (2.2–4.0)	−0.004 (0.07)	
	Quite a bit	3.5 (2.5–4.9)	0.16 (0.10)	
	Very much	3.1 (2.5–5.0)	0.17 (0.11)	0.20

β is the parameter estimate associated with the increment of one unit of ln IL-6; *IQR* Interquartile range, *SE* Standard error

For the final multivariate linear regression (Table 3), two factors (diabetes, household income) were excluded because of high collinearity with other variables. In addition, BMI at 20 years of age, TNM stage, alcohol abuse and sleep disturbance were no longer associated with IL-6. The remaining eight factors independently associated with IL-6 explained 18% of the variability of IL-6 serum level (R^2^ = 0.18). Sociodemographic characteristics positively associated with IL-6 were age (β = + 0.01, *P* <  0.0001) and male gender (β = + 0.26, *P* <  0.0005), while married patients (β = − 0.16, *P* = 0.02) had lower IL-6 levels. Three factors characterizing the patients’ medical condition were independently associated with IL-6 serum levels. Higher IL-6 serum levels were observed in patients with a lower KPS (β = + 0.38, *P* = 0.0004) and in those with a higher Charlson Comorbidity Index (β = + 0.23, *P* = 0.006 for an index of 2 or more), while IL-6 levels were lower in patients with glottic cancer (β = − 0.28, *P* <  0.0001). Two health behavior variables were positively and independently associated with IL-6 levels: current obesity (β = + 0.27, *P* = 0.0003) and tobacco consumption (β = + 0.43, *P* = 0.001 for former users; β = + 0.51, *P* = 0.0004 for current users). The bootstrap model confirmed the reliability of the final model in that the estimates of the regression coefficients and their 95% confidence intervals were very similar.

**Table 3:** Factors independently associated with IL-6 serum levels in the multivariate linear regression and in the bootstrapping regression model (*N* = 527)

Factors	Categories	Multivariate linear regression			Bootstrapping regression model		
		β (SE)	95% CI	*P*-value	β (SE)	95% CI	*P*-value
Age (years)	Continuous	0.01 (0.003)	0.01; 0.02	< 0.0001	0.01 (0.003)	0.01; 0.02	< 0.0001
Sex	Female	Reference			Reference		
	Male	0.26 (0.07)	0.12; 0.41	0.0005	0.26 (0.07)	0.12; 0.40	0.0005
Current body mass index (kg/m^2^)	< 18.5	0.08 (0.16)	−0.23; 0.38	0.63	0.07 (0.14)	−0.21; 0.38	0.63
	≥ 18.5 < 30	Reference			Reference		
	≥ 30	0.27 (0.07)	0.12; 0.41	0.0003	0.27 (0.07)	0.14; 0.40	0.0003
Marital status	Not married	Reference			Reference		
	Married	- 0.16 (0.07)	−0.29; −0.03	0.02	−0.16 (0.07)	−0.29; − 0.02	0.02
Karnofsky Performance Status (%)	90–100	Reference			Reference		
	60–80	0.38 (0.11)	0.17; 0.58	0.0004	0.37 (0.12)	0.16; 0.64	0.0004
Charlson Comorbidity Index	0	Reference			Reference		
	1	0.15 (0.07)	0.01; 0.28	0.03	0.15 (0.08)	0.01; 0.31	0.03
	≥ 2	0.23 (0.08)	0.07; 0.40	0.006	0.23 (0.07)	0.10; 0.39	0.006
Glottic cancer	No	Reference			Reference		
	Yes	−0.28 (0.06)	−0.40; −0.16	< 0.0001	− 0.28 (0.06)	− 0.41; − 0.16	< 0.0001
Tobacco consumption (all tobacco products)	Never	Reference			Reference		
	Former	0.43 (0.13)	0.17; 0.69	0.001	0.43 (0.11)	0.21; 0.63	0.001
	Current	0.51 (0.14)	0.23; 0.79	0.0004	0.51 (0.12)	0.26; 0.74	0.0004

β is the parameter estimate associated with the increment of one unit of ln IL-6; *CI* Confidence interval, *SE* Standard error

Associations and tests for linear trend between lifetime cigarette consumption and IL-6 serum levels are shown in Table 4. IL-6 serum levels increased with longer duration of cigarette smoking (*P*-value for trend = 0.03) and the number of pack-years consumed (*P*-value for trend = 0.03). Among former cigarette smokers, IL-6 levels decreased with the number of years since quitting (*P*-value for trend = 0.02).

**Table 4:** Multivariate analyses showing lifetime cigarette consumption variables associated with IL-6 serum levels (*N* = 527)

Factors	Categories	*N* (%)	Median IL–6 (ng/L) (IQR)	Adjusted β^*^ (SE)	*P*-value
Duration of cigarette smoking (years)	Never smokers	30 (5.7)	2.2 (1.0–3.3)	Reference	
	Ever smokers				
	< 32	120 (22.8)	2.6 (1.5–3.7)	0.36 (0.13)	0.008
	≥ 32 < 40	116 (22.0)	3.0 (2.2–4.6)	0.48 (0.13)	0.0004
	≥ 40 < 47	131 (24.9)	3.3 (2.5–4.3)	0.50 (0.13)	0.0001
	≥ 47	130 (24.7)	3.7 (2.7–5.4)	0.57 (0.13)	< 0.0001
			*P-*trend < 0.0001		
			*P-*trend 0.03 (excluding never smokers)		
Average number of cigarettes per day	Never smokers	30 (5.7)	2.2 (1.0–3.3)	Reference	
	Ever smokers				
	< 20	119 (22.6)	2.9 (2.2–4.3)	0.40 (0.13)	0.003
	≥ 20 < 25	70 (13.3)	3.0 (2.1–4.3)	0.52 (0.14)	0.0003
	≥ 25 < 30	167 (31.7)	3.3 (2.4–4.6)	0.54 (0.13)	< 0.0001
	≥ 30	141 (26.8)	3.2 (2.3–4.9)	0.49 (0.13)	0.0002
			*P-*trend 0.003		
			*P-*trend 0.25 (excluding never smokers)		
Pack-years of cigarettes	Never smokers	30 (5.7)	2.2 (1.0–3.3)	Reference	
	Ever smokers				
	< 32	123 (23.3)	2.7 (2.0–4.0)	0.35 (0.13)	0.008
	≥ 32 < 47	125 (23.7)	3.0 (2.3–4.3)	0.52 (0.13)	< 0.0001
	≥ 47 < 67	123 (23.3)	3.3 (2.4–4.7)	0.55 (0.13)	< 0.0001
	≥ 67	126 (23.9)	3.5 (2.5–5.2)	0.52 (0.13)	< 0.0001
			*P-*trend 0.0002		
			*P-*trend 0.03 (excluding never smokers)		
Years since cigarette smoking cessation	≥ 10	107 (20.3)	2.8 (2.0–4.3)	Reference	
	≥ 1 < 10	60 (11.4)	3.2 (2.4–4.9)	0.21 (0.08)	0.010
	< 1	201 (38.1)	3.0 (2.3–4.4)	0.20 (0.11)	0.060
			*P-*trend 0.02		

β is the parameter estimate associated with the increment of one unit of ln IL-6; *IQR* Interquartile range, *SE* Standard error. * β ajusted for all factors in Table 3, except for tobacco consumption

## Discussion

This study showed that eight factors were independently associated with circulating IL-6, a marker of HNC patients’ prognosis. IL-6 serum levels were higher with increasing age, in males, in unmarried patients, in patients with lower KPS or higher Charlson Comorbidity Indexes, and in those with HNC at a site other than the glottis. Two health behavior factors, obesity and smoking status, were independently associated with IL-6. In addition, we showed that lifetime cigarette consumption was a strong predictor of pro-inflammatory IL-6 serum levels in HNC patients with a dose-response relationship.

This cross-sectional study, based on baseline data collected at the time of randomization in a clinical trial, has several strengths and limitations. Our study population was derived from a representative population of patients with stage I and II HNC treated by radiation therapy, since 85% of the eligible HNC patients agreed to participate in this multicenter trial conducted in the province of Quebec. Only 2.4% of the participants were not included in these analyses because no serum samples were available. The bootstrapping method used for validation of the model identifying eight independent factors showed that the model was reliable in predicting circulating IL-6. To corroborate our findings, we also examined the relationships between IL-6 and long-term alcohol and tobacco exposure. To our knowledge, this is the first study conducted on HNC patients that has examined the associations between long-term exposure to alcohol and tobacco and IL-6. In cross-sectional studies, associations with lifetime exposure could be affected to some extent by patients’ ability to recall their past exposure. This potential source of bias was probably minimized in our study, since past exposure was assessed independently of the patients’ IL-6 status using a well-structured interview conducted by trained research nurses. In addition, the associations with long-term exposure to tobacco or alcohol corroborated well with those found with their respective current exposure.

High IL-6 serum levels have consistently been reported with advancing age [28] and, to a lesser extent, in middle-aged subjects selected from general populations [29, 30]. IL-6 serum levels might reflect a dysregulation of the immune system in older people but could also act as a disease marker. Ferrucci et al. [30] reported that IL-6 serum levels increased linearly with age in 367 men and 731 women aged 20–102 years from a general population. However, when cardiovascular disease, cancers and other conditions were taken into account, the magnitude of the association between age and circulating IL-6 decreased substantially but remained statistically significant in men. A recent study conducted among 987 healthy and non-obese subjects aged 20 to 80 showed that after adjusting for sex, BMI, tobacco and alcohol status, IL-6 levels in both serum and peripheral blood mononuclear cells were higher only in subjects aged 65 to 80 [31]. In our HNC population that consisted mostly of middle-aged and older people as well as in Duffy et al.’s HNC study population [19], age remained positively associated with IL-6 serum levels after adjustment for comorbidities and several risk factors, suggesting a true association with age. However, residual confounding could have occurred in these studies, since part of the age association with IL-6 in HNC patients could be due to the presence of occult second primary cancer at the time of HNC diagnosis, a condition associated with increased IL-6 serum levels [14].

Recent studies, using Mendelian randomization analyses, conducted among thousands of people, have established a causal relationship between genetic markers of adiposity and circulating IL-6 levels [32–34]. The BMI-genetic scores were consistently and positively associated with IL-6, but the associations were attenuated in younger subjects and in men [32]. Among HNC patients, we also found a positive association between IL-6 and obesity, as defined by a BMI ≥ 30 kg/m^2^. Adipose tissue contains multiple types of cells, including adipocytes, macrophages and immune active cells, all metabolically active in producing IL-6, especially in the context of obesity [35, 36]. Studies have suggested that IL-6 could be released differentially according to body fat distribution patterns [37]. Adipose tissue biopsies done in 47 healthy overweight or obese subjects showed that pro-inflammatory T-helper (Th)-1, Th17 and CD8 T-cells were significantly more frequent in visceral adipose tissue (VAT) than in subcutaneous adipose tissue (SAT) [38]. Concurrently, IL-6 expression, a marker of Th1 and M1 macrophage activation, was twofold greater in VAT than in SAT. In 97 subjects aged 22–69, randomly selected from the general population, VAT and SAT were quantified using a two-dimensional ultrasound image [39]. IL-6 serum levels were positively associated with VAT in overweight/obese participants, while there was no association with SAT. This differential expression of IL-6 according to the VAT and SAT compartments might partly explain the discordance between studies of the effect of sex on circulating IL-6 [29, 30, 40]. Large studies conducted in general populations in the United States showed that, compared with women, men have a higher volume of VAT [41] and a lower volume of SAT [42].

In our study population of early-stage HNC, site was a stronger predictor of IL-6 serum levels than TNM stage. This is explained well by other studies. Elevated serum levels of IL-6 are consistently observed among patients with advanced-stage HNC, in particular in those with positive lymph nodes [19, 43]. In patients with early-stage (T1-T2, N0, M0) laryngeal cancer, those with glottic cancer have significantly lower IL-6 serum levels compared with those with supraglottic cancer, while there was no difference between laryngeal subsites in advanced stages [44]. In our study, 50% of the patients had stage I glottic cancer, which is an infiltrating tumor limited to the vocal cords and frequently no more than a few millimeters in size. Using high throughput technologies, several gene signatures and protein networks have been identified in HNC [45]. The IL-6/IL-6R/JAK/STAT3 is one of these pathways, which contributes to the development and progression of HNC cancers [45, 46]. The IL-6 gene is also a target of nuclear factor–kappa B (NF-kB) [45, 47]. NF-kB inhibition downregulates IL-6 gene and protein expression and decreases the release of several cytokines. In addition, there is a cross-talk between NF-kB and STAT3 signaling pathways in HNC cell lines. Experimental studies are currently being conducted to identify selective inhibitors of IL-6 induced STAT3 in the treatment of HNC [46, 48, 49].

Human Papilloma Virus (HPV) infection is recognized to be a risk factor for specific HNC sites, in particular for oropharyngeal cancer [50]. In our cohort of early HNC, most of the patients had laryngeal cancer (83.0%), while 11.7% had oral cancer and only 3.2% had oropharyngeal cancer. A large international study using 3680 samples, estimated that the prevalence of HPV-DNA, targeting 25 HPV types, was only 5.7% in laryngeal cancer, which represents the majority of our study population [51]. For oral cavity cancer, the HPV-DNA prevalence was 7.4%, while for oropharyngeal cancers, the prevalence increased over calendar time. During the time-period 1995–1999, corresponding to the recruitment of the majority of our HNC cohort, HPV-DNA prevalence was 10.1%. We could reasonably conclude that a small number of patients in our cohort were HPV-positive. In HNC, few studies have evaluated the association between HPV infection and circulating IL-6. These studies, conducted among small series of HNC patients, gave conflicting results. Guerrera et al. [52] found that IL-6 serum levels were significantly higher among HPV-negative patients compared to HPV-positive patients, while Argiris et al. [53] reported no difference in serum IL-6 levels between HPV-positive and negative patients.

In our study, married patients showed lower IL-6 levels compared to non-married patients. It is doubtful that this relationship could be attributable to HPV infection since the opposite would be expected, according to the findings of Guerrera et al. [52]. One plausible explanation is that married people have better dietary and other health behaviours associated with lower levels of circulating cytokines [54]. Overall, these data suggest that HPV status probably had little impact on IL-6 status in this HNC cohort.

Modifiable health behaviors have been reported to influence systemic IL-6 levels [17–19]. Some authors have hypothesized that IL-6 levels might differ according to sex because of different health behaviors [29]. Large studies conducted in general populations have consistently reported a U- or J-shaped association between alcohol intake and IL-6 serum levels with a nadir for moderate consumption (1–2 drinks per day) [55–58]. In particular, a recent prospective cohort study conducted among 8209 British civil servants showed that moderate drinkers (8 to 168 g of ethanol per week for men and 8 to 112 g for women) stable over a period of 10 years had lower serum levels of IL-6 compared with stable non-drinkers and stable heavy drinkers [55]. Current and past alcohol consumption were not predictors of IL-6 serum levels in our study, but most of the patients had low and moderate alcohol consumption. As reported by Duffy et al. [19], we found that only patients with a history of alcohol abuse had higher IL-6 serum levels, but this association was no longer statistically significant after adjustment for other covariates, including tobacco.

Activated monocytes/macrophages [59–61] and epithelial cells [62] have been described as producing pro-inflammatory cytokines, including IL-6. These cell types can be activated by carcinogens found in tobacco products [63, 64]. In a mouse model, cigarette smoke has been shown to upregulate IL-6, leading to the differentiation of T-helper cells in IL-17-producing T-cells [65]. In addition, tobacco-induced oxidative stress also activates the NF-kB family of transcription factors, which upregulates the expression of pro-inflammatory cytokines [66, 67]. In our study, current smoking status was a health behavior independently associated with IL-6 serum levels. This corroborates well with the findings of the only cross-sectional study previously conducted on HNC patients [19] as well as with studies conducted on healthy people [68]. In addition, our study showed that cumulative exposure to cigarette smoking was significantly associated in a dose-dependent manner with IL-6 serum levels. This supports a causality link between smoking and IL-6 status in HNC patients. Due to the impact of tobacco on IL-6 serum levels found in this HNC population and our previous findings showing that IL-6 serum level was a strong predictor of SPC in HNC patients [14], further analyses will be conducted to examine whether the association between tobacco and SPC is partly mediated by IL-6. This will contribute to a better understanding of the role of inflammation in the occurrence of cancer.
